# Swedish exchange students’ alcohol use, drug use, risky sexual behaviour, mental health, and self-rated health: A follow-up study

**DOI:** 10.1177/14550725231160331

**Published:** 2023-03-15

**Authors:** Emil Danehorn, Marie Oscarsson, Goldina Smirthwaite, Ulla Peterson, Katarina Swahnberg

**Affiliations:** Department of Health and Life Sciences, Linnaeus University, Kalmar, Sweden; Department of Health and Life Sciences, Linnaeus University, Kalmar, Sweden; Faculty of Arts and Social Sciences, Karlstad University, Karlstad, Sweden; Department of Health and Life Sciences, Linnaeus University, Kalmar, Sweden; Department of Health and Life Sciences, Linnaeus University, Kalmar, Sweden

**Keywords:** alcohol use, drug use, exchange students, mental health, sexually risky behaviour

## Abstract

**Aims:** To follow up on exchange students’ alcohol use, drug use, mental health, self-rated health, and risky sexual behaviour after a semester abroad and to compare them with students who remained on campus. **Methods:** The study design was a follow-up study based on a previous baseline survey of 114 prospective exchange students and 451 campus students. Of the original 565 students, 48 (42.1%) prospective exchange students and 209 (43.3%) campus students responded to the follow-up. Both the baseline survey and the follow-up survey included the General Health Questionnaire 12, one single item from Self-Rated Health, and nine items from Knowledge, Attitudes and Sexual Behaviour in Young People in Sweden. **Results:** We found a statistically significant increase in the weekly consumption of alcohol among exchange students after their semester abroad. A larger proportion of exchange students had sex with a new partner and sex with more than three partners during their semester abroad compared to follow-up campus students. **Conclusions:** Our findings indicate that exchange students consume alcohol more frequently during their semester abroad and indulge in sexually risky behaviour. Exchange students’ use of alcohol and sexually risky behaviour could be associated with even greater risks due to them being in an unknown environment, unfamiliar culture, and with limited support from family and friends. This highlights the need for further research on exchange students’ experiences, especially concerning alcohol use and sex while abroad.

Being able to return from a semester abroad with new experiences and knowledge is probably the expectation of many exchange students. A semester abroad can contribute to and increase knowledge about other cultures and to a general development in tolerance and understanding; however, the transition to a new environment might not always be an easy endeavour (Brown & Holloway, 2008).

Exchange students may experience psychological strain while abroad, such as homesickness and/or loneliness, and the level of homesickness is often higher in the beginning of the semester and wears off as time passes (Götz et al., 2019). Loneliness can be enhanced if the exchange students are having difficulties making new friends or maintaining relationships back home, for example, due to time differences (Ng et al., 2018). It has been suggested that another strong contributor to loneliness while being abroad is difficulties in communicating with others. When language skills increase, it becomes easier to interact and participate in various events, which means that the feelings of loneliness and exclusion might decrease (Brown & Holloway, 2008). Being able to speak the local language has been shown to be more important than reading or writing (Sherry et al., 2010). The need to readapt when returning home can also lead to feelings of isolation, deprivation, and psychological strain, and this has been shown to be prominent in exchange students who have spent at least 6 months abroad (Dettweiler et al., 2015).

Previous research has shown that university students who experience psychological strain also use alcohol and cannabis to a higher degree as a coping strategy (Deasy et al., 2014). In addition, a reoccurring phenomenon in students’ social life is to play various drinking games, where students are encouraged and prompted to drink large amounts of alcohol, and drinking games and binge-drinking have been associated with unprotected sex and waking up unsure if sex had occurred (Simmons et al., 2005). Generally, it seems as though university studies increase the opportunity for sexual encounters, predominantly due to students being young and being around the same age and having impulsive behaviour, reduced parental influence, and an unawareness or denial of risks, all of which can lead to casual and unprotected sex (Chanakira et al., 2014). This could be considered a sexually risky behaviour, which is often defined as sex with multiple partners, sex under the influence of drugs/alcohol, and/or sex without protection (Tikkanen et al., 2011b)

Risky alcohol consumption, on the other hand, is often difficult to discern because there are several variables to consider, such as age, sex, underlying diseases, and pregnancy. However, in general, the risk for ill-health or negative consequences, such as accidents or putting oneself into dangerous situation due to a decrease in judgement, increases with the consumption of 10 or more standard glasses of alcohol per week, or more than four standard glasses at the same occasion (Allebeck et al., 2018). Approximately 16% of the Swedish population as a whole, and 21% of Swedish youths aged 16–29 years consume alcohol at a risky level. According to the Public Health Agency of Sweden, alcohol consumption is one of the most prominent causes for a premature death, thus having a major negative impact on public health (Public Health Agency of Sweden, 2021).

There are supposedly several motives for why students frequently consume alcohol, for instance, social contexts, such as student gatherings, and/or to enhance their mood (Chanakira et al., 2014) or to cope with psychological strain (Deasy et al., 2014). It has been shown that students who frequently consume large quantities of alcohol have a higher risk of developing mental ill-health and to experience reduced satisfaction with life (Jensen et al., 2021). Students’ high consumption of alcohol also negatively affects their academic performance to a high degree because it can lead to missed classes and concentration difficulties (Tembo et al., 2017).

University students in general are a well-researched group when it comes to health, sexually risky behaviour, and alcohol use; however, exchange students are not well studied, especially whether or how their health or behaviour changes during their semester abroad. It is possible that the experience of being an exchange student in an unfamiliar environment, with unforeseen expectations, has a negative effect on their health.

## Aim

The aim of this study was to follow up on exchange students’ alcohol use, drug use, mental health, self-rated health, and risky sexual behaviour after a semester abroad and to compare them with students who remained on campus.

## Methods

### Sample

The sample consisted of Swedish university students who had spent a semester abroad as exchange students as well as a comparison group of students who remained on campus. Both groups participated in two web surveys, one before departure and one after approximately 6 months. The first survey served as a baseline and the second as a follow-up. A total of 2,298 students were asked to participate in the baseline, of which 383 were students who intended to spend a semester abroad, hereafter referred to as prospective exchange students, and 1,915 students who remained on campus forming a comparison group, hereafter referred to as campus students. All prospective exchange students at a university and five times as many randomly selected campus students aged above 18 years in semesters 4 and 5 were asked to participate via email.

### Procedure

An online survey along with information about the aim of the study, that participation was voluntary, and how data were going to be handled was sent to the baseline groups at two different occasions, in May and November 2018. All students who responded to the baseline survey received a follow-up survey after approximately 6 months.

### Measures

The baseline survey and the follow-up survey included the General Health Questionnaire 12 (GHQ12) (Sconfienza, 1998), one single item from the Short Form Health Survey (SF-36), Self-Rated Health (SRH) (Lundin & Dalman, 2020), and nine items from Knowledge, Attitudes and Sexual Behaviour in Young People in Sweden (UngKAB)

We measured mental health with the GHQ12, which was created to identify anxiety, depression, and stress reactions (Lundin & Dalman, 2020). The GHQ12 comprises 12 questions about mental health concerning the following: Enjoying day-to-day activities; Losing sleep over worry; Being able to concentrate; Being capable of making decisions; thinking of oneself as worthless; being under constant strain; playing a useful part in things; not being able to overcome difficulties; facing up to problems; feeling unhappy and depressed; losing confidence in oneself; and being reasonably happy. The questions could be answered with the options “Better than usual”, “Same as usual”, “Worse than usual”, or “Much worse than usual”, which were given scores of 0–3, respectively. Of the 12 questions, six were negatively worded. GHQ12 was in the range of 0–36, and a lower score indicate better mental health. Cronbach’s alpha was 0.88 for male students and 0.90 for female students.

One item of the SF-36 was used to measure SRH. The SF-36 was previously validated in the general Swedish population, and the instrument is used to investigate bodily functions of both a physical and psychological nature (Sullivan et al., 1995). The students were asked to rate their health as excellent, very good, good, fair, or bad. Excellent, very good, and good were interpreted as good health, while fair and bad were considered poor health.

UngKAB was used to investigate drug use, alcohol use, and sexual behaviour (Tikkanen et al., 2011a). UngKAB was originally developed by the Social Health Department in Sweden and is used as an online survey to measure the life situation of Swedish men and women aged 15–29 years. We used the following items from UngKAB: use of cannabis, use of other drugs, frequency of alcohol consumption, standard glasses of alcohol per occasion, sex with a new partner, and alcohol together with sex during the semester abroad or in the past 6 months, as well as the total number of sexual partners. Sex with a new partner and alcohol also included a follow-up question on whether protection from sexually transmitted infections had been used. The questions about drugs and alcohol could be answered with daily, a few times a week, a few times a month, more rarely, and never, as well as how many standard glasses of alcohol were consumed on a single occasion. Questions about sex had numerical or yes/no answers.

#### Statistical analysis

Descriptive statistics used SPSS version 25 (IBM Corp., Armonk, NY, USA). We used a 95% confidence interval (CI) without a correction for continuity to determine the significant differences between independent proportions in UngKAB and SRH (Wilson, 1927). The Mann–Whitney U test was used to determined the significant differences between the mean scores on the GHQ12.

#### Ethical consideration

To ensure the well-being of the participants, we provided contact information to the student health department and various independent support organisations, as well as all members of the research group. The instructions for the questionnaire and the attached letter contained detailed information on the content of the survey, the aim of the study, and that participation was voluntary and anonymous. Because detailed information was provided, we assumed that those who, for any reason, did not want or could not participate in the study abstained. Submitting the web survey was considered giving informed consent. We carried out the study in accordance with relevant ethical principles and guidelines of the Declaration of Helsinki. This protocol was approved by Regional Ethical Board in Linköping (Dnr 2017/504-3).

## Results

The total response rate for the two initial baseline surveys was 24.8% (n = 570). In May 2018, 24.5% (n = 49) of the prospective exchange students and 23.1% (n = 231) of the campus students responded. In November 2018, 36.1% (n = 66) of the prospective exchange students and 24.5% (n = 224) of the campus students responded. Out of the 570 students, five had selected “other gender”, and they were excluded because they were too few to form a separate group. In the two follow-up surveys, the total response rate was 45.5% (n = 257), and 34.7% (n = 17) of the prospective exchange students and 48.5% (n = 112) of the campus students responded to the May survey follow-up. For the November survey follow-up, 47% (n = 31) of the exchange students and 43.3% (n = 97) of the campus students responded. The mean age was 24.6 years for prospective exchange students, 23.4 years for follow-up exchange students, 27.6 years for campus students, and 26.6 for follow-up campus students.

**Figure 1.**
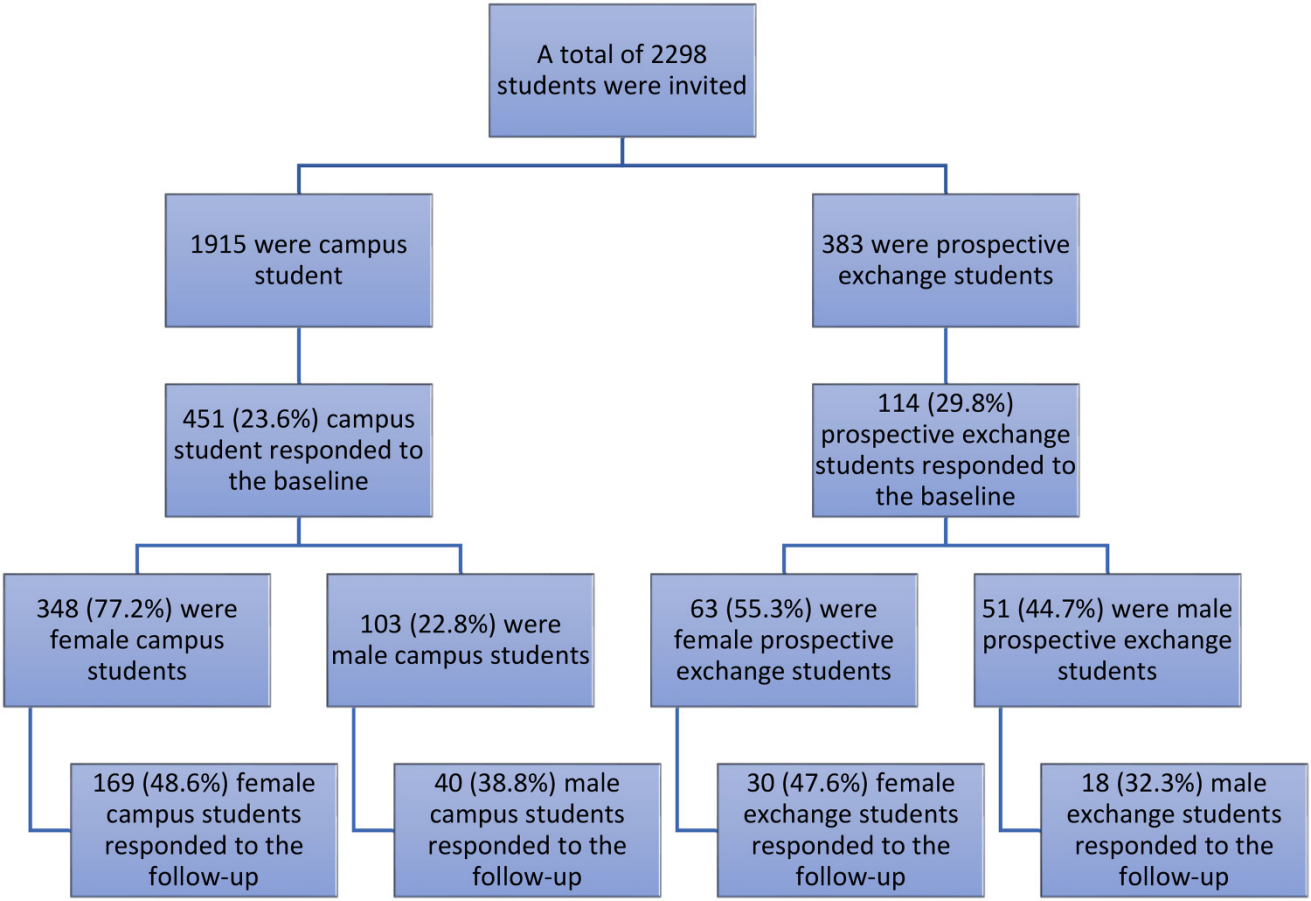
Male and female exchange and campus students invited to participate at baseline and follow-up.

The distribution between male/female prospective exchange students who completed the survey was 44.7/55.3% with a mean age of 25/24.3 years. For exchange students, the distribution between male/female was 37.5/62.5% with a mean age of 25.3/23.9 years. For campus students, the distribution between male/female was 22.8/72.2% with a mean age of 26.4/28 years. For follow-up campus students, the distribution between male/female was 19.1/80.9% with a mean age of 26.3/27.4 years (Figure 1).

### Mental health

At baseline, prospective exchange students as a group rated their mental health as being better (GHQ12 = 11.2) than that of campus students (GHQ12 = 13.3) (p = .00). In the follow-up survey, exchange students (GHQ12 = 9.6) also rated their mental health as being better than that of campus students (GHQ12 = 12.3) (p = .05), and follow-up campus students rated their mental health as better (GHQ12 = 12.3) after 6 months compared to baseline campus students (GHQ12 = 13.3) (p = .02). There were no significant differences in mental health for exchange students after a semester abroad.

### Self-rated health

At baseline, there were no statistically significant differences in self-rated health between the groups (Table 1). At follow-up, exchange students rated their health as fair (7%) to a lower degree than campus students (16%). More exchange students rated their health as very good (52.1%) compared to follow-up campus students (35.9%). There were no statistically significant differences in SRH for exchange students and neither for campus students after 6 months (Table 2).

**Table 1.** Comparison of prospective exchange students’/campus students’ and exchange students’/follow-up campus students' self-rated health, drug, and alcohol use.

Prospective exchange students		Campus students		Exchange students	Follow-up campus students	
	n = 114	n = 451	Δ (95% CI)	n = 48	n = 209	Δ (95% CI)
Self-rated health						
Excellent	14 (12.3)	36 (8.0)	−4.3 (–1.3, 11.9)	6 (12.5)	14 (6.7)	−5.8 (–2.1, 18.3)
Very good	52 (45.6)	165 (36.6)	−9.0 (–0.9, 19.1)	**25** (**52.1)**	**75** (**35.9)**	**−16.2** (**0.9, 31.0)**
Good	39 (34.2)	164 (36.4)	+2.2 (−7.9, 11.4)	15 (31.3)	77 (36.8)	+5.6 (–9.8, 18.7)
Fair	**8** (**7.0)**	**72** (**16.0)**	**+9** (**2.0, 14.0)**	**1** (**2.1)**	**39** (**18.7)**	**+16.6** (**6.6, 22.7)**
Bad	1 (0.9)	14 (3.1)	+2.2 (–1.9, 4.4)	1 (2.1)	4 (1.9)	−0.2 (–3.2, 9.1)
Used cannabis in the past 6 months						
Daily	1 (0.9)	3 (0.7)	−0.2 (–1.3, 4.2)	0 (0.0)	1 (0.5)	+0.5 (–6.9, 2.7)
A few times a week	**3** (**2.6)**	**1** (**0.2)**	**−2.4** (**0.4, 7.2)**	1 (2.1)	0 (0.0)	−2.1 (–0.4, 10.9)
A few times a month	0.0	5 (1.1)	+1.1 (–2.2, 2.6)	0 (0.0)	1 (0.5)	+0.5 (–6.9, 2.7)
More rarely	**16** (**14.0)**	**27** (**6.0)**	**−8.1** (**2.2, 15.8)**	7 (14.6)	18 (8.6)	−6.0 (–2.7, 18.9)
Never	**94** (**82.5)**	**415** (**92.0)**	**+9.6** (**3.0, 17.9)**	40 (83.3)	189 (90.4)	+7.1 (–2.2, 20.4)
Other drugs in the past 6 months						
Daily	0 (0.0)	1 (0.2)	+0.2 (–3.0, 1.3)	0 (0.0)	0 (0.0)	
A few times a week	0 (0.0)	0 (0.0)		1 (2.1)	0 (0.0)	−2.1 (–0.4, 10.9)
A few times a month	**3** (**2.6)**	**0** (**0.0)**	**−2.6** (**0.7, 7.5)**	0 (0.0)	0 (0.0)	
More rarely	5 (4.4)	11 (2.4)	−2.0 (–1.2, 7.5)	2 (4.2)	8 (3.8)	−0.3 (–4.3, 10.3)
Never	**106** (**93.0)**	**439** (**97.3)**	**+4.4** (**0.4, 10.7)**	45 (93.8)	201 (96.2)	+2.4 (–3.0, 13.2)
Alcohol consumption in the past 6 months						
Daily	0 (0.0)	0 (0.0)		1 (2.1)	0 (0.0)	−2.1 (–0.4, 10.9)
A few times a week	25 (21.9)	106 (23.5)	+1.6 (–7.6, 9.4)	**27** (**56.3)**	**43** (**20.6)**	**−35.7** (**20.5, 49.6)**
A few times a month	44 (38.6)	167 (37.0)	−1.6 (–8.0, 11.7)	13 (27.1)	71 (34.0)	+6.9 (–8.3, 19.3)
More rarely	37 (32.5)	126 (27.9)	−4.5 (–4.5, 14.4)	**5** (**10.2)**	**59** (**28.2)**	**+17.8** (**4.8, 26.6)**
Never	8 (7.0)	52 (11.5)	+4.5 (–2.2, 9.3)	**2** (**4.2)**	**36** (**17.2)**	**+13.1** (**2.3, 19.5)**
Standard glasses of alcohol						
**1**–**2**	**8** (**7.0)**	**132** (**29.3)**	**+22.3** (**14.9, 27.8)**	**7** (**14.6)**	**77** (**36.8)**	**+22.3** (**8.2, 32.2)**
**3**–**4**	**17** (**14.9)**	**119** (**26.4)**	**+11.5** (**2.9, 18.3)**	18 (37.5)	52 (24.9)	−12.6 (–1.2, 27.7)
**5**–**6**	**41** (**36.0)**	**98** (**21.7)**	**−14.3** (**5.1, 24.0)**	11 (22.9)	42 (20.1)	−2.8 (–8.5, 17.3)
**7**–**9**	**28** (**24.6)**	**47** (**10.4)**	**−14.1** (**6.5, 23.1)**	8 (16.7)	18 (8.6)	−8.1 (–1.1, 21.3)
≥**10**	**12** (**10.5)**	**15** (**3.3)**	**−7.2** (**2.3, 14.3)**	2 (4.2)	3 (1.4)	−2.7 (–1.3, 12.6)
Does not drink alcohol	8 (7.0)	40 (8.9)	+1.9 (–4.8, 6.4)	2 (4.2)	17 (8.1)	+4 (–6.3, 9.4)

*Note.* Values are given as n (%) unless otherwise indicated. CI = confidence interval. Bold numbers indicate a significant difference. ▹ indicates a proportional difference. +/- indicates increased/decreased proportional changes.

**Table 2.** Exchange students’ and campus students' self-rated health, drug, and alcohol use before and after a semester abroad or <6 months.

Prospective exchange students		Exchange students		Campus students	Follow-up campus students	
	n = 114	n = 48	Δ (95% CI)	n = 451	n = 209	Δ (95% CI)
Self-rated health						
Excellent	14 (12.3)	6 (12.5)	+0.2 (–9.6, 13.3)	36 (8.0)	14 (6.7)	−1.3 (−3.5, 5.2)
Very good	52 (45.6)	25 (52.1)	+6.5 (–10.0, 22.6)	165 (36.6)	75 (35.9)	−0.7 (–7.3, 8.4)
Good	39 (34.2)	15 (31.3)	−3.0 (–13.2, 17.5)	164 (36.4)	77 (36.8)	+0.5 (–7.3, 8.5)
Fair	8 (7.0)	1 (2.1)	−4.9 (–4.5, 11.4)	72 (16.0)	39 (18.7)	+2.7 (–3.3, 9.3)
Bad	1 (0.9)	1 (2.1)	+1.2 (–3.1, 10.1)	14 (3.1)	4 (1.9)	−1.2 (–2.0, 3.5)
Used cannabis in the past 6 months						
Daily	1 (0.9)	0 (0.0)	−0.9 (–6.6, 4.8)	3 (0.7)	1 (0.5)	−0.2 (–2.0, 1.5)
A few times a week	3 (2.6)	1 (2.1)	−0.6 (–8.4, 5.7)	1 (0.2)	0 (0.0)	−0.2 (–1.6, 1.3)
A few times a month	0 (0.0)	0 (0.0)		5 (1.1)	1 (0.5)	−0.6 (–1.6, 2.1)
More rarely	16 (14.0)	7 (14.6)	+0.6 (–1.0, 14.2)	27 (6.0)	18 (8.6)	+2.6 (–1.4, 7.6)
Never	94 (82.5)	40 (83.3)	+0.9 (–13.3, 12.2)	415 (92.0)	189 (90.4)	−1.6 (–2.8, 6.8)
Other drugs in the past 6 months						
Daily	0 (0.0)	0 (0.0)		1 (0.2)	0 (0.0)	−0.2 (–1.6, 1.3)
A few times a week	0 (0.0)	1 (2.1)	+2.1 (–1.6, 10.9)	0 (0.0)	0 (0.0)	
A few times a month	3 (2.6)	0 (0.0)	−2.6 (–5.0, 7.5)	0 (0.0)	0 (0.0)	
More rarely	5 (4.4)	2 (4.2)	−0.2 (–10.0, 6.5)	11 (2.4)	8 (3.8)	+1.4 (–1.3, 5.1)
Never	106 (93.0)	45 (93.8)	+0.8 (–10.4, 8.2)	439 (97.3)	201 (96.2)	−1.2 (–1.5, 4.9)
Alcohol consumption in the past 6 months						
Daily	0 (0.0)	1 (2.1)	+2.1 (–1.6, 10.9)	0 (0.0)	0 (0.0)	
A few times a week	**25** (**21.9)**	**27** (**56.3)**	**+34.3** (**18.0, 49.0)**	106 (23.5)	43 (20.6)	−2.9 (–4.1, 9.4)
A few times a month	44 (38.6)	13 (27.1)	−11.5 (–4.6, 25.5)	167 (37.0)	71 (34.0)	−3.1 (–4.9, 10.7)
More rarely	**37** (**32.5)**	**5** (**10.2)**	**−22** (**7.9, 32.8)**	126 (27.9)	59 (28.2)	+0.3 (–6.8, 7.9)
Never	8 (7.0)	2 (4.2)	−2.9 (–7.5, 9.8)	**52** (**11.5)**	**36** (**17.2)**	**+5.7** (**0.1, 12.0)**
Standard glasses of alcohol						
**1**–**2**	8 (7.0)	7 (14.6)	+7.6 (–2.1, 20.6)	132 (29.3)	77 (36.8)	+7.6 (–0.0, 15.4)
**3**–**4**	**17** (**14.9)**	**18** (**37.5)**	**+22.6** (**8.1, 37.7)**	119 (26.4)	52 (24.9)	−1.5 (–5.9, 8.4)
**5**–**6**	41 (36.0)	11 (22.9)	−13.1 (–2.9, 26.3)	98 (21.7)	42 (20.1)	−1.6 (–5.3, 8.0)
**7**–**9**	28 (24.6)	8 (16.7)	−7.9 (–6.8, 19.7)	47 (10.4)	18 (8.6)	−1.8 (–3.4, 6.2)
≥**10**	12 (10.5)	2 (4.2)	−6.4 (–4.4, 14.0)	15 (3.3)	3 (1.4)	−1.9 (–1.1, 4.2)
Does not drink alcohol	8 (7.0)	2 (4.2)	−2.9 (–7.5, 9.8)	40 (8.9)	17 (8.1)	−0.7 (–4.3, 5.0)

*Note.* Values are given as n (%) unless otherwise indicated. CI = confidence interval. Bold numbers indicate a significant difference. ▹ indicates a proportional difference. +/- indicates increased/decreased proportional changes.

### Alcohol and drug use

At baseline, there was no statistically significant difference between prospective exchange students and campus students regarding alcohol consumption during the last 6 months. However, prospective exchange students drank proportionally more standard glasses of alcohol compared to campus students (Table 1). At follow-up, a larger proportion of the exchange students (56.3%) consumed alcohol a few times per week compared with follow-up campus students (20.6%) (Table 2). In addition, a larger proportion follow-up campus student had not consumed alcohol in the last 6 months (17.2%) compared to exchange students (4.2%).

Exchange students increased their weekly alcohol consumption from 21.9% to 56.3% while the rare use of alcohol decreased from 32.5% to 10.2% (Table 2).

### Sexual behaviour

At baseline, a larger proportion campus student (12.4%) had no sexual partners in the last 6 months compared to prospective exchange students (4.4%). In addition, a larger proportion of prospective exchange students (40.4%) had sex with a new partner in the past 6 months compared to campus students (21.3%). In addition, a larger proportion of prospective exchange students (70.2%) had sex under the influence of alcohol in the last 6 months compared to campus students (53.4%) (Table 3). At follow-up, a larger proportion exchange student (47.9%) reported that they had sex with a new partner during the last 6 months than follow-up campus students (21.1%). Further, a larger proportion exchange student had sex with more than three partners during the last 6 months (16.7%) than follow-up campus students (3.8%). A larger portion (21.5%) of follow-up campus students had had no sexual partners in the last 6 months compared to campus students (12.4%). There were no statistically significant differences in sexual behaviour for exchange students after a semester abroad (Table 4).

**Table 3.** Comparison of prospective exchange students’/campus students’ and exchange students’/follow-up campus students' sexual behaviour.

Prospective exchange students		Campus students		Exchange students	Campus students follow-up	
	n = 114	n = 451	Δ (95% CI)	n = 48	n = 209	Δ (95% CI)
No. of sexual partners in the past 6 months						
Never had sex	11 (9.6)	39 (8.6)	−1.0 (–4.1, 8.2)	2 (4.2)	13 (6.2)	+2.1 (–8.1, 7.2)
None	**5** (**4.4)**	**56** (**12.4)**	**+8** (**1.9, 12.2)**	5 (10.4)	45 (21.5)	+11.1 (–1.7, 19.6)
**1**–**3**	73 (64.1)	330 (73.2)	+9.1 (–0.1, 19.1)	33 (68.8)	143 (68.4)	−0.3 (–15.0, 13.4)
**>3**	**25** (**21.9)**	**26** (**5.8)**	**−16.2** (**9.1, 24.8)**	**8** (**16.7)**	**8** (**3.8)**	**−12.8** (**4.1, 25.9)**
Sex with new partner in the past 6 months						
Yes	**46** (**40.4)**	**96** (**21.3)**	**−19.1** (**9.6, 28.9)**	**23** (**47.9)**	**44** (**21.1)**	**−26.9** (**12.1, 41.5)**
No	**68** (**59.6)**	**355** (**78.7)**	**+19.1** (**9.6, 28.9)**	**25** (**52.1)**	**165** (**78.9)**	**+26.8** (**12.1, 41.5)**
STI protection while having sex with a new partner						
Yes	24 (52.2)	44 (45.8)	−6.3 (–10.9, 23.1)	16 (69.6)	24 (54.5)	−15.0 (–9.6, 35.8)
No	22 (47.8)	52 (54.2)	+6.3 (–10.9, 23.1)	7 (30.4)	20 (45.5)	+15.0 (–9.6, 35.8)
Alcohol together with sex during exchange trip or in the past 6 months						
Yes	**80** (**70.2)**	**241** (**53.4)**	**−16.7** (**6.7, 25.6)**	29 (60.4)	104 (49.8)	−10.7 (–5.0, 24.9)
No	**34** (**29.8)**	**210** (**46.6)**	**+16.7** (**6.7, 25.6)**	19 (39.6)	105 (50.2)	+10.7 (–5.0, 24.9)
STI protection while having sex under the influence of alcohol						
Yes	29 (36.3)	68 (28.2)	−8.0 (–3.4, 20.2)	**14** (**48.3)**	29 (27.9)	**−20.4** (**1.1, 39.3)**
No	51 (63.7)	173 (71.8)	+8.0 (–3.4, 20.2)	**15** (**51.7)**	75 (72.1)	**+20.4** (**1.1, 39.3)**

*Note.* Values are given as n (%) unless otherwise indicated. CI = confidence interval; STI = sexually transmitted infection. Bold numbers indicate a significant difference. ▹ indicates a proportional difference. +/- indicates increased/decreased proportional changes.

**Table 4.** Exchange students’ and campus students' sexual behaviour before and after a semester abroad or <6 months.

Prospective exchange students		Exchange students		Campus students	Campus students follow-up	
	n = 114	n = 48	Δ (95% CI)	n = 451	n = 209	Δ (95% CI)
No. of sexual partners in the past 6 months						
Never had sex	11 (9.6)	2 (4.2)	−5.5 (–5.2, 12.9)	39 (8.6)	13 (6.2)	−2.4 (–2.3, 6.3)
None	5 (4.4)	5 (10.4)	+6.0 (–2.0, 18.0)	**56** (**12.4)**	**45** (**21.5)**	**+9.1** (**3.1, 15.8)**
**1 to 3**	73 (64.1)	33 (68.8)	+4.7 (–11.6, 19.3)	330 (73.2)	143 (68.4)	−4.8 (–2.6, 12.4)
**>3**	25 (21.9)	8 (16.7)	−5.3 (–9.2, 16.9)	26 (5.8)	8 (3.8)	−1.9 (–2.0, 5.1)
Sex with new partner in the past 6 months						
Yes	46 (40.4)	23 (47.9)	+7.6 (–8.7, 23.8)	96 (21.3)	44 (21.1)	−0.2 (–6.8, 6.6)
No	68 (59.6)	25 (52.1)	−7.6 (–8.7, 23.8)	355 (78.7)	165 (78.9)	+0.2 (–6.8, 6.6)
STI protection while having sex with a new partner						
Yes	24 (52.2)	16 (69.6)	+17.4 (–7.2, 37.8)	44 (45.8)	24 (54.5)	+8.7 (–8.9, 25.5)
No	22 (47.8)	7 (30.4)	−17.4 (–7.2, 37.8)	52 (54.2)	20 (45.5)	−8.7 (–8.9, 25.5)
Alcohol together with sex during exchange trip or in the past 6 months						
Yes	80 (70.2)	29 (60.4)	−9.8 (–5.7, 25.8)	241 (53.4)	104 (49.8)	−3.7 (–4.5, 11.8)
No	34 (29.8)	19 (39.6)	+9.8 (–5.7, 25.8)	210 (46.6)	105 (50.2)	+3.7 (–4.5, 11.8)
STI protection while having sex under the influence of alcohol						
Yes	29 (36.3)	14 (48.3)	+12.0 (–8.1, 31.8)	68 (28.2)	29 (27.9)	−0.3 (–10.4, 10.1)
No	51 (63.7)	15 (51.7)	−12.0 (–8.1, 31.8)	173 (71.8)	75 (72.1)	+0.3 (–10.4, 10.1)

*Note.* Values are given as n (%) unless otherwise indicated. CI = confidence interval; STI = sexually transmitted infection. Bold numbers indicate a significant difference. ▹ indicates a proportional difference. +/- indicates increased/decreased proportional changes.

## Discussion

We found a statistically significant proportional increase in the weekly consumption of alcohol among exchange students, whereas the alcohol consumption of campus students did not increase between the two measurements. Exchange students rated their health and mental health as being better than that of campus students both before and after their semester abroad, but there were no significant changes in exchange students’ health and mental health before or after their semester abroad. A larger proportion exchange student had sex with a new partner and had sex with more than three partners compared to follow-up campus students.

A larger proportion of both prospective and exchange students had sex with a new partner and had sex with more than three partners during the last 6 months compared to campus students. Exchange students’ sexual activity can be construed as a sexually risky behaviour (Tikkanen et al., 2011a) and the alcohol consumption can be considered risky as several exchange students consumed alcohol a few times a week and/or more than four standard glasses on a single occasion (Allebeck et al., 2018). It is possible that the high intake of alcohol and higher frequency of sexual encounters among exchange students is connected, and previous research suggests that using alcohol while traveling as a tourist is associated with increased casual sexual encounters (Downing et al., 2010). The high alcohol consumption is also in line with previous research. European exchange students have a higher level of alcohol consumption than local students, both in general use and in terms of binge drinking (Dormal et al., 2019). In addition, exchange students experience more negative alcohol-related consequences, such as blackout drinking, than campus students (Aresi et al., 2016). Several predictors for heavy drinking have been identified when it comes to exchange students. Students from the United States who, before they leave for exchange studies, express intentions to get involved in student activities and who travel to urban European cities have been shown to have increased alcohol consumption (Pedersen et al., 2014). Protective factors against heavy drinking are, among others, living with a native host family and studying the native language and local culture (Pedersen et al., 2014).

On the other hand, the semester abroad did not seem to have a significant effect on mental health for exchange students even though they transitioned to a new environment and their consumption of alcohol increased, which is associated with decreased mental health for students (Jensen et al., 2021). The unchanged mental health and SRH could be due to high self-esteem, which has previously been associated with Swedish exchange students (Petersson et al., 2016). High self-esteem has been shown to be a protective factor against mental disorders such as anxiety and depression (Henriksen et al., 2017). However, high self-esteem also tends to increase the desire to experiment with sex and alcohol (Baumeister et al., 2003).

There were several parameters that were not significantly changed after a semester abroad; however, it should be noted that an unchanged parameter is also a valid finding. For instance, that mental health had not been affected for the exchange student in this study might suggest that there is no immediate need for interventions to improve mental health. On the other hand, exchange students’ increased use of alcohol and their sexually risky behaviour could be associated with even greater risks due to them being in an unknown environment with limited support from family and friends. There were tendencies, although not statistically significant, that exchange students reduced their sexually risky behaviour while abroad, but still at a higher level than follow-up campus students. There are also tendencies of decreased heavy drinking, but not statistically significant. However, the response rate was low, therefore the results should be interpreted with caution.

## Methodological consideration

Our findings should be viewed with an understanding of its limitations, especially the representability of Swedish exchange students. The distribution of responding male/female students and mean age was relatively similar throughout the study. This could suggest that the sample is representative, to some degree, for Swedish exchange students, which increases the credibility of this study. However, the sample size was small and greatly reduced from the baseline to the follow-up, it is possible that students with common characteristics were more prone to answer the survey, causing a sampling bias and limiting the representativeness (Jacobsen, 2021). As Swedish exchange students are a highly select group (Petersson et al., 2016), it is difficult to assess the level of representativeness, which makes generalisation challenging (Polit & Beck, 2020).

The source population, i.e., exchange students, was limited; therefore, we used comparison groups to increase the credibility of the study and to strengthen the results (Bruchmann, 2017). Moreover, more female students in all groups responded to the survey, and the female response rate for the follow-up was higher compared to male students. As the comparisons have been made group-wise with no regard to sex, this may have affected the results.

The instruments used in the study have been previously used with similar populations (Petersson et al., 2016), and all questions have been satisfactorily answered and there was no internal loss. Cronbach’s alpha for the GHQ12 was high, which indicates good reliability. The response rate was low; however, the interest in answering epidemiological surveys is generally low and has declined in recent years (Galea & Tracy, 2007). Specific factors for this study that could have affected the response rate are that the first survey was time-consuming and that the questions might have felt intrusive (Simmons & Swahnberg, 2019). As the source population was limited in the baseline survey, greatly reduced in the follow-up, and prospective exchange students had approximately 50 countries to choose from, we decided not to collect data on which country the students had visited, as that could have made it possible to reveal the student's identities. Therefore, this study does not consider the difference in drinking cultures of the receiving country, which makes it difficult to draw conclusions on what caused the increased alcohol consumption. There were also a few wide confidence intervals, especially for alcohol consumption, which increases the uncertainty of the results, and this could be due to the small sample size.

## Conclusion

Our findings indicate that exchange students consume alcohol more frequently during their semester abroad and indulge in sexually risky behaviour. Exchange students’ use of alcohol and sexually risky behaviour could be associated with even greater risks due to them being in an unknown environment, unfamiliar culture, and with limited support from family and friends. This highlights the need for further research on exchange students’ experiences, especially concerning alcohol use and sex while abroad.
